# Circular RNA PRKCI promotes glioma cell progression by inhibiting microRNA-545

**DOI:** 10.1038/s41419-019-1863-z

**Published:** 2019-08-13

**Authors:** Xuebang Zhang, Han Yang, Lihao Zhao, Gang Li, Yuxia Duan

**Affiliations:** 1 Department of Chemoradiation Oncology, The first affiliated hospital of Wenzhou Medical University, Wenzhou, Zhejiang P.R. China; 2 Department of Radiology, The First Affiliated Hospital of Wenzhou Medical University, Wenzhou, Zhejiang P.R. China

**Keywords:** Targeted therapies, Oncogenes

## Abstract

We here tested expression and potential functions of circular RNA PRKCI (circPRKCI) in human glioma. Our results show that circPRKCI is upregulated in human glioma tissues and glioma cells, correlating with downregulation of its potential target, microRNA-545 (miR-545). In A172 and primary human glioma cells, shRNA-mediated silencing of circPRKCI inhibited cancer cell growth, survival, proliferation, and migration. Conversely, ectopic circPRKCI overexpression promoted A172 cell progression. miR-545 is the primary target of circPRKCI in glioma cells. Forced overexpression of miR-545 mimicked circPRKCI shRNA-induced actions, inhibiting glioma cell survival and proliferation. In contrast, miR-545 inhibition, by a lentiviral antagomiR-545 construct, reversed circPRKCI shRNA-induced anti-A172 cell activity. Importantly, neither circPRKCI shRNA nor circPRKCI overexpression was effective in miR-545-knockout (Cas9 method) A172 cells. Importantly, the subcutaneous and orthotopic A172 xenograft growth was significantly inhibited by circPRKCI silencing. Collectively, circPRKCI promotes human glioma cell progression possibly by inhibiting miR-545. Targeting circPRKCI-miR-545 cascade could efficiently inhibit human glioma cells.

## Introduction

Glioma, the most common brain tumor and a global health threat, causes significant mortalities each year^1–3^. Current therapeutic options for glioma have failed to substantially improve patients’ prognosis^4–6^. High-grade (grade III-IV) glioma (i.e., glioblastoma) has one of the worst survival among all malignancies^1–3^, which is possibly due to its molecular heterogeneity^1^. It is therefore urgent to further explore the pathological mechanisms for glioma progression, which could help to develop novel and more efficient molecularly-targeted therapies^7–9^.

microRNA (miRNAs), long non-coding RNAs (LncRNAs), and circular RNAs (circRNAs) are non-coding RNAs^10,11^. Recent studies have proposed the pivotal roles of these non-coding RNAs in glioma progression^12^. Unlike other non-coding RNAs, circRNAs can form highly stable circular structure via joining of the 3′ and 5′ terminals^11,13,14^. circRNAs function as miRNAs sponges, regulating gene expression in cancer cells^11,14,15^. Dysregulation of circRNAs is often detected in glioma^12^.

Qiu et al. have recently discovered a novel and oncogenic circRNA, namely circRNAs PRKCI (circPRKCI)^16^. circPRKCI, with 1,484-bp long, is back-spliced of two exons (15 and 16) of *PRKCI* gene located at 3q26.2^16^. circPRKCI is upregulated in lung adenocarcinoma in part due to the amplification of 3q26.2 locus, promoting cancer cell proliferation and tumorigenesis^16^. circPRKCI is mainly present in the cytoplasm, sponging miR-545 and miR-589, thereby abolishing the suppressing of their target, the transcription factor *E2F7*^16^. Shi et al. have shown that circPRKCI promotes AKT3 expression and esophageal squamous cell proliferation by sponging miR-3680-3p^17^. In human hepatocellular carcinoma (HCC) cells circ-PRKCI functions as the sponge of miR-545, promoting cell survival^18^.The expression and potential functions of circPRKCI in human glioma cells are tested in the present study.

## Materials and methods

### Chemicals and reagents

The antibodies utilized in this study were purchased from Abcam (Cambridge, MA). The reagents for cell culture were purchased from Hyclone (Logan, UT). Puromycin, polybrene and all other chemicals were provided by Sigma–Aldrich (St. Louis, Mo). All primers, sequences, virus, and expression constructs were designed, provided, and sequence verified by Shanghai Genechem Co. (Shanghai, China).

### Cell culture

The established A172 glioma cells were provided by Dr. Cao^19^. Brain cortical tissues were obtained from 15-week-old normal fetal brains (prepared from Dr. Zhang^20^), dissociated, digested, and filtered as previously described^21,22^. The dissociated cells were centrifuged and resuspended in MEM medium with applied supplements^22^. Cells were seeded at a density of 2 × 10^6^ cells/mL on poly-lysine-coated tissue culture flasks. For neuronal culture, astrocytes were limited by 4-day treatment with FDU (Sigma). Neuronal cultures were grown for eight days (day in vitro 8, DIV8) before any further experiments. The primary human astrocytes (95% positive for glial fibrillary acidic protein) were provided from Dr. Cao^19^, prepared from 12-week cortical tissues from normal fetal brains. All procedures were approved by the Ethics Committee of Wenzhou Medical University.

### Primary human glioma cells and tissues

In this study the primary human glioma cells were provided by Dr. Cao^19,23^, derived from three written-informed consent glioma patients, named as “Pri-1/-2/-3”. Primary human glioma cells were cultured in complete RPMI medium with necessary supplements and antibiotics^24,25^. The human glioma tissues and paired paracancer normal brain tissues were again provided by Dr. Cao^19,23^, stored in liquid nitrogen and subjected to further biomedical analyses. The protocols of this study were approved by the Ethics Committee of Wenzhou Medical University, in according to Declaration of Helsinki.

### Quantitative real-time PCR (qPCR)

Total cellular and tissue RNAs were isolated by the Trizol reagent (Invitrogen, Grand Island, NY) and quantified. mRNA expression was tested by using the SYBR GREEN PCR Master Mix (Applied Biosystems, Beijing, China) under an ABI 7600 fast Real-time PCR System (Applied Biosystems). Relative expression of targeted mRNAs was calculated by 2^−ΔΔCt^ method, using *GAPDH* as the internal control. circPRKCI and miR-545 levels were tested by the TransStartTM SYBR Green qPCR Supermix (TransGen Biotech, Beijing, China), using U6 small nuclear RNA as the internal control. All the primers were listed in Table. 1.

**Table. 1:** Primer sequences of the study

Genes	Forward primer	Reverse primer
*circPRKCI*	5′-ATTCAGGGACACCCGTTCTT-3′	5′-CTCTTCAGAACACTTGCAGCTT-3′
*U6*	5′-CTCGCTTCGGCAGCACA-3′	5′-AACGCTTCACGAATTTGCGT-3′
*GAPDH*	5′-CGCTCTCTGCTCCTCCTGTTC-3′	5′-ATCCGTTGACTCCGACCTTCAC-3′
*E2F7*	5′-CTGCTGCGCTAGACTTGGAT-3′	5′-TCTCTTAGTAGGACCACCAACG-3′
*RIG-1*	5′-AGCACTGGGTGCATGAGGCCT-3′	5′- TCCTTTGTGGCAGCACCCAATG-3′
*miR-545*	5′-ACGGCCATACCACCCTGAAC-3′	5′-GGCGGTCTCCCATCCAAGTA-3′

### Western blotting

Equivalent amounts of total cellular lysates (40 μg per treatment) were separated by 10–12% of SDS-PAGE gels, then transferred to the polyvinylidene fluoride (PVDF) blots (Merck Millipore, Darmstadt, Germany). After blocking in 10% non-fat milk, the blots were incubated with the applied primary antibodies, followed by incubation with corresponding secondary antibodies. Antibody-antigen binding was detected by an enhanced chemiluminescence (ECL) substrate kit (Invitrogen), with the results quantified by an ImageJ software (NIH, Bethesda, MD).

### Cell Counting Kit-8 (CCK-8) assay

Cells were initially seeded into 96-well plates at 5 × 10^3^ cells per well. Following incubation for 72 h, CCK-8 solution (10 μL/well, Dojindo Molecular Technologies, Gaithersburg, MD) was added to each well. After incubation for another 3 h, CCK-8 optical density (OD) values were measured at test wavelength of 450 nm.

### Colony formation assay

A172 cells were initially seeded at 1 × 10^4^ cells per 10-cm dish. Colony formation assays were conducted for 10 days, and the colonies were fixed and stained (with 1% crystal violet solution). The number of colonies was counted manually.

### In vitro migration assay

A172 cells (1 × 10^5^ cells in 300 μL serum-free medium) were seeded into the upper part of each “Transwell” chambers (12-μm pore size, BD Biosciences, Heidelberg, Germany). The lower compartments were filled with medium with 10% FBS. Following incubation for 24 h, non-migrated cells on the upper surface were wiped out. The migrated cells, on the lower surface, were fixed and stained.

### EdU assay of proliferation

Cells were seeded into six-well plates at 6 × 10^4^ cells per well, and cultured for 48 h. An EdU (5-ethynyl-20-deoxyuridine) Apollo-567 Kit (RiboBio, Guangzhou, China) was applied. EdU and DAPI dyes were added to glioma cells for additional 4 h. Under a fluorescent microscope cell nuclei were visualized. For each condition total 300 nuclei in five random views were included to calculate EdU ratios (EdU/DAPI × 100%).

### Annexin V FACS assay

Following the applied genetic treatments, Annexin V-FITC and Propidium Iodide (PI) dyes (each at 10 μg/mL, BD Pharmingen, San Diego, CA) were added for 30 min under the dark at room temperature. Cell apoptosis was analyzed by a flow cytometry machine (Beckman Coulter, Brea, CA).

### circPRKCI shRNA

Two shRNAs targeting non-overlapping sequences (“Seq-1/2”) of circPRKCI were individually sub-cloned into GV248 (hU6-MCS-Ubiquitin-EGFP-IRES-puromycin) construct (Shanghai Genechem Co.), then transfected to HEK-293 cells with lentivirus package plasmid mix (Shanghai Genechem Co.). The generated circPRKCI shRNA lentivirus (“LV-circPRKCI shRNA”) was added to cultured glioma cells (in polybrene medium). Following selection by puromycin (5.0 μg/mL, for 4–5 passages), stable cells were established. Silencing of circPRKCI (over 90% knockdown efficiency) in stable cells was confirmed by qPCR. Control cells were transfected with lentiviral scramble control shRNA.

### Ectopic overexpression of circPRKCI or miR-545

The full-length circPRKCI^16,26^ and pri-miR-545^16,26^ were synthesized by Shanghai Genechem Co, sub-cloned to a lentiviral GV248 construct (Shanghai Genechem Co.). The construct, together with lentivirus package plasmid mix, was co-transfected to HEK-293 cells to generate circPRKCI-expressing lentivirus (“LV-circPRKCI”) and miR-545-expressing lentivirus (“LV-miR-545”). Following filtration and enrichment, LV-circPRKCI or LV-miR-545 was added to cultured glioma cells. Puromycin was added to select stable cells. Control cells were infected with lentivirus with empty vector.

### miR-545 inhibition

The miR-545 inhibitor precursor, provided by Shanghai Genechem, was sub-cloned into the GV248 vector, co-transfected into 293 T cells with lentivirus package plasmids to generate antagomiR-545 lentivirus (LV-antagomiR-545). The latter was transduced to A172 cells for 24 h. Puromycin was added again to select the stable cells. miR-545 inhibition in the stable cells was confirmed by qPCR.

### miR-545 knockout

The CRISPR/Cas9 miR-545-KO lentivirus, with sgRNA targeting the pri-miR-545 sequence, was synthesized and verified by Shanghai Genechem Co. (Shanghai, China), added to A172 cells. The infected cells were than cultured in puromycin selection medium until stable cells were achieved. Cells were further transfected with or without the lentiviral circPRKCI shRNA construct (“LV-circPRKCI shRNA”) or the lentiviral circPRKCI overexpression construct (“LV-circPRKCI”). Expression of miR-545 and circPRKCI was tested by qPCR.

### RNA immunoprecipitation (RIP)

A172 cells were lysed in complete RIP lysis buffer (purchased from Beyotime Biotechnology, Suzhou, China), and the cell extracts (800 μg lysates per treatment) were incubated with the magnetic beads conjugated with anti-Argonaute 2 (Ago2, Sigma) or control anti-IgG antibody (Sigma) for 12 h. The beads were washed and incubated with Proteinase K. Finally, the purified RNA was subjected to qPCR analysis, with results normalized to the Input control.

### A172 xenograft assay

The severe combined immunodeficient (SCID) mice (4–5 week old, about 18 g weight) were provided by the Animal Center of Suzhou University (Suzhou, China), inoculated via subcutaneous (*s.c*.) injection with five million A172 cells with circPRKCI shRNA or control shRNA, in 200 µL of Matrigel gel/basal medium. Mouse body weights and tumor volumes were measured weekly, and tumor volume calculated by a described formula^19^. For intracranial tumor implantation, A172 glioma cells (1 × 10^6^ cells) were implanted as described^27^. All animal procedures were approved by the Ethics Committee of Wenzhou Medical University.

### Statistics

Statistical analyses were performed by SPSS 21.0 software (SPSS Inc., Chicago, IL). All data were presented as mean ± standard deviation (SD). Statistical differences were performed by one-way ANOVA within multiple comparisons with post hoc Bonferroni test. To determine significance between two treatment groups, the two-tailed *t*-tests were carried out. *P* values < 0.05 were considered statistically significant.

## Results

### circPRKCI is upregulated in human glioma tissues and cells

**Fig. 1:**
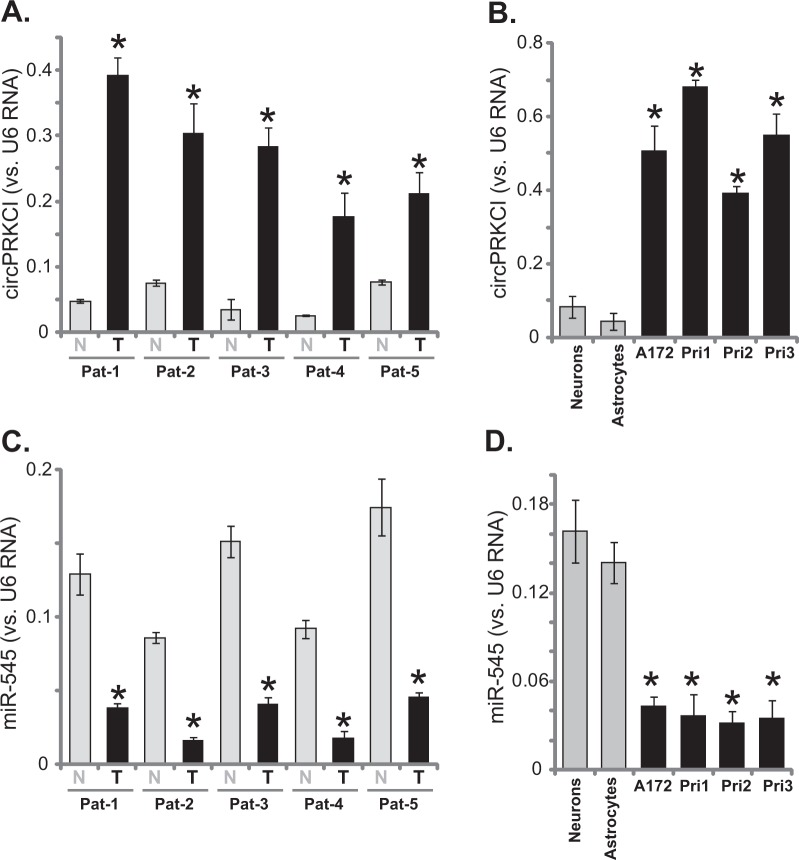
circPRKCI is upregulated in human glioma tissues and cells. Total RNA was extracted from the described human tissues and cells, expression of circPRKCI (**a**, **b**) and miR-545 (**c**, **d**) was tested by qPCR assays, results were normalized to *U6 RNA*. “Pat” stands for glioma patient number. Error bars stand for mean ± standard deviation (SD, *n* = 5). *P < 0.05 vs. “N” tissues (**a**, **c**) or primary neuronal culture (“Neurons”) (**b**, **d**)

First, circPRKCI expression in human glioma tissues was examined. A total of five pairs of fresh glioma tissues (“T”, from Dr. Cao^19^) and parecancer normal brain tissues (“N”) were obtained. qPCR assays were performed to test circPRKCI expression. Results, in Fig. 1a, demonstrated that circPRKCI levels are significantly upregulated in all tested glioma tissues, when compared its levels in the normal brain tissues. Furthermore, circPRKCI is upregulated in A172 glioma cells and in the primary human glioma cells (“Pri-1/-2/-3”, see Methods) (Fig. 1b). While its levels are low in primary human neuronal cultures and human astrocytes (Dr. Cao^19^) (Fig. 1b).

It has been previously shown that circPRKCI functions as the sponge of miR-545, a tumor-suppressive miRNA^16^. We therefore tested miR-545 expression in glioma tissues and cells. As demonstrated, miR-545 levels are significantly downregulated in glioma tissues (Fig. 1c), as well as in the established and primary human glioma cells (Fig. 1d). In contrast, miR-545 expression is relatively high in normal brain tissues, primary human neuronal cultures and human astrocytes (Fig. 1c, d). Another important miRNA target of circPRKCI, miR-589^16^, was not detected in our system. These results show that circPRKCI is upregulated in human glioma tissues and cells, correlating with miR-545 downregulation.

### circPRKCI shRNA inhibits A172 glioma cell growth, survival, proliferation, and migration

**Fig. 2:**
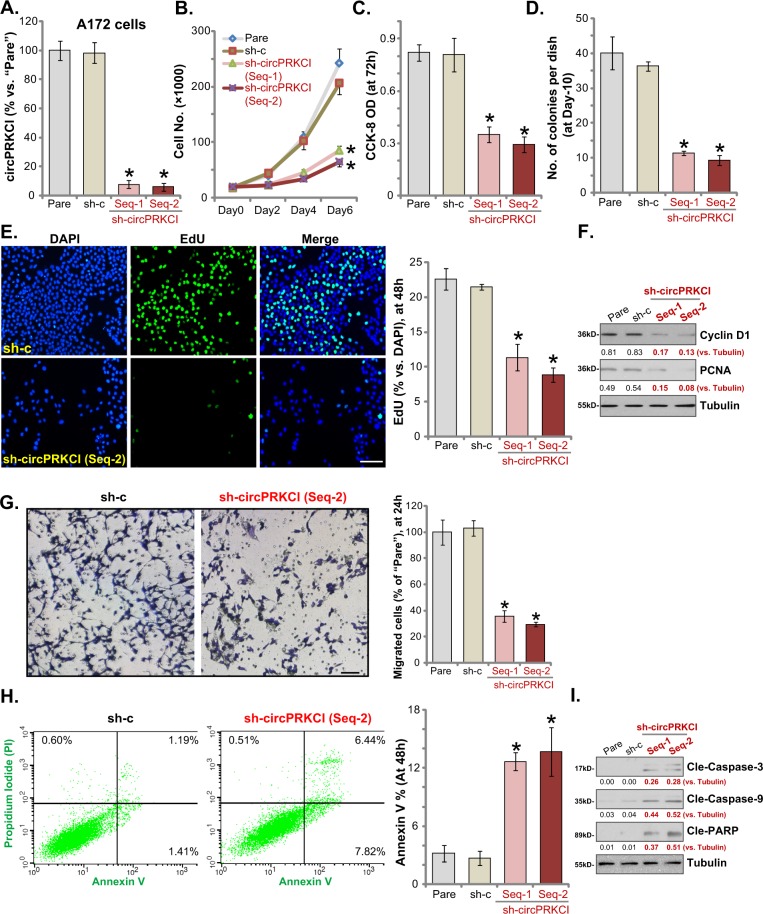
circPRKCI shRNA inhibits A172 glioma cell growth, survival, proliferation and migration. A172 glioma cells were transduced with GV248 lentiviral circPRKCI shRNA (“Seq-1/Seq-2”, non-overlapping sequence) construct or scramble non-sense control shRNA (“sh-c”) construct for 24 h, stable cells were then established via selection with puromycin-containing medium. circPRKCI expression was tested by qPCR (**a**); Cell growth (**b**), viability (**c**), proliferation (**d**–**f**), migration (**g**), and apoptosis (**h**, **i**) were tested by appropriate assays mentioned in the text. For all the functional assays, the exact same amount of viable cells were plated into each well/dish initially (at 0 h) (Same for all Figures). “Pare” stands for the parental control cells. Error bars stand for mean ± standard deviation (SD, *n* = 5). *P < 0.05 vs. “Pare” cells. Experiments in this figure were repeated four times, and similar results were obtained. Bar = 100 μm (**e**, **g**)

In order to study the potential function of circPRKCI in glioma cells, a shRNA strategy was applied. GV248 lentiviral shRNA, targeting non-overlapping sequence (“Seq-1/Seq-2”) against circPRKCI (“sh-circPRKCI”), was added to A172 glioma cells. Following puromycin selection stable cells were established. Testing circPRKCI expression in the stable cells, by qPCR assays, confirmed that circPRKCI levels decreased over 90% (vs. the parental control cells) (Fig. 2a). By counting cell number, we show that circPRKCI shRNA-expressing A172 cells grew significantly slower than the control cells (Fig. 2b). Cell viability, or the CCK-8 OD, was decreased as well by circPRKCI shRNA (Fig. 2c). Further experimental results demonstrated that circPRKCI shRNA decreased the number of A172 cell colonies (Fig. 2d) and EdU incorporation (Fig. 2e), indicating its anti-proliferative activity. Expression of proliferation marker proteins, including cyclin D1 and proliferating cell nuclear antigen (PCNA), was significantly downregulated as well in circPRKCI-silenced A172 glioma cells (Fig. 2f).

“Transwell” assay was performed to test cell migration in vitro. Results demonstrated that following circPRKCI silencing, the number of migrated A172 cells was significantly reduced (Fig. 2g). Additional studies showed that circPRKCI shRNA induced apoptosis activation in A172 cells, evidenced by increased Annexin V staining (Fig. 2h) as well as cleavages of caspase-3, caspase-9 and PARP [poly (ADP-ribose) polymerase] (Fig. 2i). The non-sense scramble control shRNA lentivirus (“sh-C”), as expected, exerted no significant effect on circPRKCI expression (Fig. 2a) nor A172 cell functions (Fig. 2b–i). Collectively, these results show that circPRKCI silencing inhibited cell growth, survival, proliferation, and migration, whiling inducing apoptosis activation in A172 glioma cells.

### circPRKCI silencing inhibits primary human glioma cell progression

**Fig. 3:**
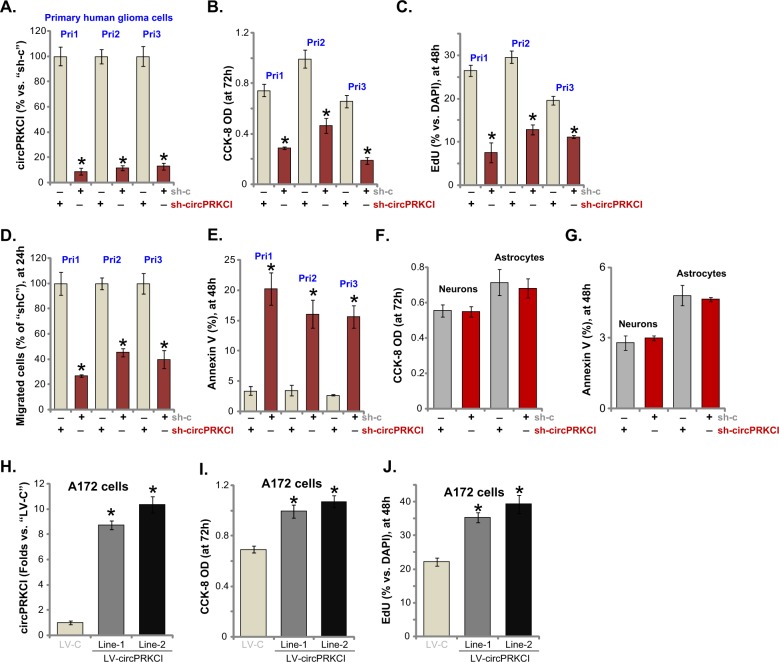
circPRKCI silencing inhibits primary human glioma cell progression. The primary human glioma cells, derived from three different patients (“Pri-1/−2/−3”), as well as primary human neuronal cultures (“Neurons”) and primary human astrocytes (“Astrocytes”), were transduced with GV248 lentiviral circPRKCI shRNA (“Seq-1”) or scramble non-sense control shRNA (“sh-c”) for 24 h, stable cells were established via selection with puromycin. circPRKCI expression was tested by qPCR (**a**); Cell viability (CCK-8 OD, **b**, **f**), proliferation (EdU ratio, **c**), migration (“Transwell” assay, **d**), and apoptosis (Annexin V ratio, **e**, **g**) were tested. A172 cells were transduced with lentiviral circPRKCI expression construct (“LV-circPRKCI”) or empty vector (“LV-C”) for 24 h, stable cells were established via puromycin selection; circPRKCI expression (**h**), cell viability (**i**), and proliferation (**j**) were tested similarly. Error bars stand for mean ± standard deviation (SD, *n* = 5). *P < 0.05 vs. “sh-c”/“LV-C” cells. Experiments in this figure were repeated four times, and similar results were obtained

Next, we studied the potential function of circPRKCI in primary cells. The primary human glioma cells, derived three different patients (“Pri-1/-2/-3”, see Fig. 1), were transduced with lentiviral circPRKCI shRNA (“Seq-1”, see Fig. 2). Following selection by puromycin stable cells were established, showing 80–90% reduction of circPRKCI expression (Fig. 3a). shRNA-induced knockdown of circPRKCI in the primary glioma cells induced significant viability reduction (Fig. 3b), proliferation inhibition (Fig. 3c), cell migration inhibition (Fig. 3d) and apoptosis activation (Fig. 3e). Importantly, circPRKCI shRNA failed to affect the viability (Fig. 3f) and apoptosis (Fig. 3g) in primary human neuronal cultures (“Neurons”) and primary human astrocytes (“Astrocytes”), where circPRKCI levels are low (Fig. 1), indicating a cancer cell specific effect by circPRKCI.

Next, a lentiviral circPRKCI expression construct (“LV-circPRKCI”) was transduce to A172 cells. Two lines of stable cells, “Line-1” and “Line-2”, were established with selection by puromycin, showing 8–10 folds increase of circPRKCI expression (Fig. 3h). LV-circPRKCI-expressing cells presented with increased cell viability (CCK-8 OD, Fig. 3i) and proliferation (EdU ratio, Fig. 3j), as compared to the control A172 cells with empty vector. Therefore, forced overexpression of circPRKCI promoted A172 cell progression, further confirming its oncogenic role in glioma cells.

### miR-545 is the primary target of circPRKCI in glioma cells

**Fig. 4:**
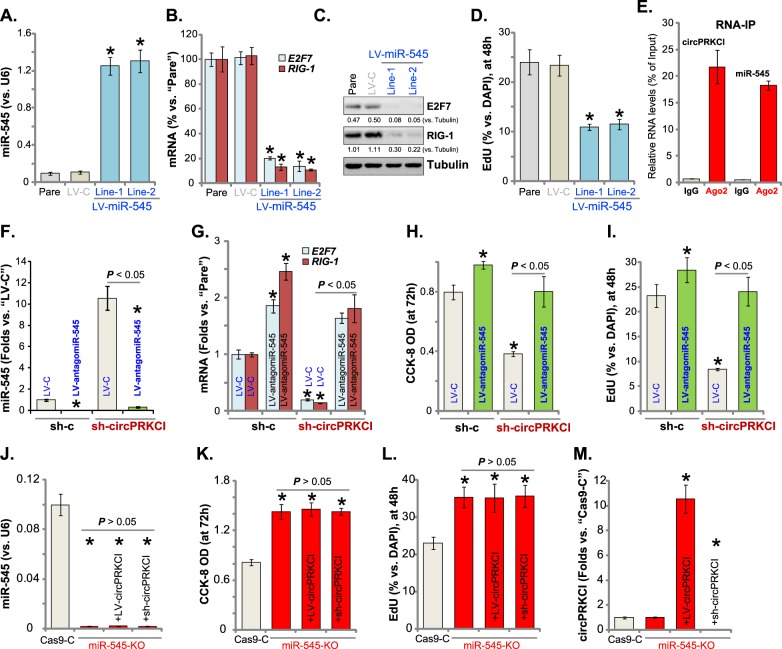
miR-545 is the primary target of circPRKCI in glioma cells. A172 glioma cells were transfected with lentiviral pri-miR-545 construct (“LV-miR-545”) or empty vector with scramble control microRNA (“LV-C”), stable cells were established via puromycin selection. Expression of miR-545 (**a**, qPCR), listed mRNAs (**b**, by qPCR), and proteins (**c**, by Western blotting assay) was shown; Cell proliferation (**d**, EdU ratio) was tested. RNA immunoprecipitation (RIP) assay results confirmed that Argonaute 2 (Ago2) immunoprecipitated with both circPRKCI and miR-545 in A172 glioma cells (**e**). Stable A172 cells, with lentiviral circPRKCI shRNA (“Seq-1”) or scramble non-sense control shRNA (“sh-c”), were further transfected with a lentiviral miR-545 inhibitor construct (“LV-antagomiR-545”) or LV-C, miR-545 (**f**), listed mRNAs (**g**), cell viability (**h**), and proliferation (**i**) were tested similarly. A172 glioma cells were transfected with lentiviral CRISPR-Cas9-pri-miR-545-KO construct (“miR-545-KO”) or CRISPR-Cas9 control construct (“Cas9-C”), stable cells were established via puromycin selection; miR-545-KO cells were further transfected with the lentiviral circPRKCI shRNA (“sh-circPRKCI”, “Seq-1”) or the lentiviral circPRKCI expression construct (“LV-circPRKCI”), miR-545 expression (**j**), cell viability (**k**), proliferation (**l**), and circPRKCI expression (**m**) were tested. “Pare” stands for the parental control cells. Error bars stand for mean ± standard deviation (SD, *n* = 5). *P < 0.05 vs. “Pare”/“sh-c”/“Cas9-C” cells. Experiments in this figure were repeated four times, and similar results were obtained

It has been shown that circPRKCI sponges miR-545^16^, a tumor-suppressive microRNA^16,28,29^. We first explored the potential function of miR-545 in glioma cells. A lentiviral pri-miR-545 construct (LV-miR-545) was transfected to A172 cells. Following selection by puromycin two stable cell lines (“Line-1/-2”) were established, showing significantly increased miR-545 expression (Fig. 4a). Expression of miR-545 targets, including the transcription factor *E2F7* and *RIG-1* (*retinoic acid-inducible gene-I*)^16,28^, was largely downregulated (Fig. 4b, c). Forced expression of miR-545 inhibited A172 cell proliferation (EdU ratio, Fig. 4d), mimicking actions by circPRKCI shRNA (Fig. 2).

The Argonaute 2 (Ago2) protein can bind to both circRNAs and miRNAs, initiating the ceRNA mechanism and miRNA sponging^16,30–32^. In A172 glioma cells the RIP assay results revealed that circPRKCI and miR-545 were both efficiently pulled down by the anti-Ago2 antibody, but not by the non-specific anti-IgG antibody (Fig. 4e). This is consistent with previous findings in lung adenocarcinoma cells^16^. Indeed, in the circPRKCI shRNA-expressing A172 cells, miR-545 levels were significantly increased (Fig. 4f), causing *E2F7* and *RIG-1* downregulation (Fig. 4g). Significantly, inhibition of miR-545, by a lentiviral miR-545 inhibitor construct (“LV-antagomiR-545”) (Fig. 4f), completely reversed *E2F7* and *RIG-1* inhibition by circPRKCI shRNA (Fig. 4g). Significantly, in A172 cells circPRKCI shRNA-induced viability reduction (Fig. 4h) and proliferation inhibition (Fig. 4i) were nullified by LV-antagomiR-545. LV-antagomiR-545 by itself enhanced *E2F7*/*RIG-1* expression (Fig. 4g), A172 cell viability (Fig. 4h) and proliferation (Fig. 4i). These results indicate that circPRKCI possibly sponges tumor-suppressive miR-545 in A172 cells. Conversely, circPRKCI shRNA inhibited A172 cell progression by restoring miR-545 expression.

To further confirm that miR-545 is the primary target of circPRKCI, the CRISPR/Cas9 method was applied to completely and stably knockout pri-miR-545 in A172 cells (Fig. 4j). As compared to the control cells, miR-545-KO A172 cells showed increased cell viability (Fig. 4k) and proliferation (Fig. 4l). Importantly, neither LV-circPRKCI nor circPRKCI shRNA was effective in the miR-545-KO cells (Fig. 4k, l), although both did significantly change circPRKCI expression (Fig. 4m). These results confirm that miR-545 is the primary target of circPRKCI in mediating its actions in glioma cells.

### circPRKCI silencing inhibits subcutaneous A172 glioma growth in SCID mice

**Fig. 5:**
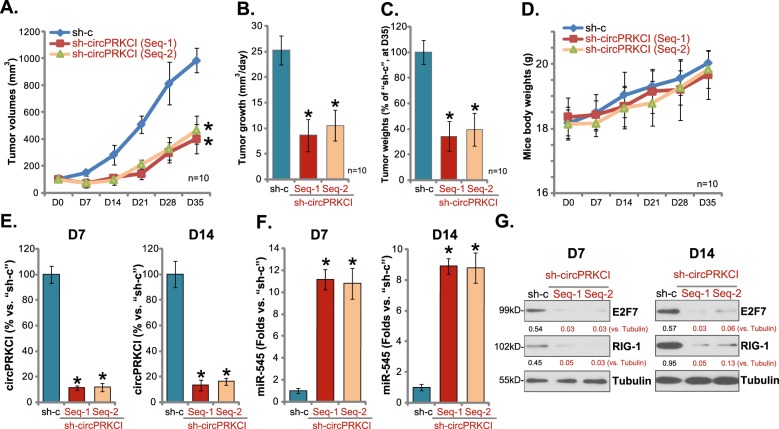
circPRKCI silencing inhibits subcutaneous A172 glioma growth in SCID mice. Weekly tumor growth curve of subcutaneous A172 gliomas with circPRKCI shRNA (“Seq-1/Seq-2”) or scramble non-sense control shRNA (“sh-c”) was shown (**a**); Estimated daily tumor growth was calculated using the described formula (**b**); At D35, tumors of all three groups were isolated and weighted (**c**); Mice body weights were recorded (**d**). At D7 and D14, one tumor of each group was isolated (total six tumors). Each tumor was randomly cut into five small pieces (*n* = 5), individually dissolved into the lysis buffer. Expression of circPRKCI (**e**), miR-545 (**f**), and listed proteins (**g**) in each piece was tested separately. Results were integrated and statistics analyses were performed (**e**, **f**). Representative blotting data were shown (**g**). Error bars stand for mean ± standard deviation (SD). *P < 0.05 vs. “sh-c” tumors

To study the potential function of circPRKCI in vivo, stable A172 glioma cells, with circPRKCI shRNA (“Seq-1/Seq-2”) or scramble non-sense control shRNA (“sh-c”), were *s.c*. injected to the flanks of SCID mice. Within 3 weeks the subcutaneous A172 tumors were established, with tumor volumes close to 100 mm^3^ (labeled as Day-0/“D0”). Recording weekly tumor volumes, in Fig. 5a, demonstrated that A172 xenografts with circPRKCI shRNA grew significantly slower than the control tumors (with “sh-c”). When calculating the estimated daily tumor growth, using the formula [tumor volume at Day-35 (D35) subtracting tumor volume at Day-0 (D0)] ÷ 35, we showed again that subcutaneous A172 tumor growth was significantly inhibited by circPRKCI shRNA (Fig. 5b). At D35, tumors of all three groups were isolated and weighted. Results confirmed that circPRKCI shRNA-expressing A172 tumors weighted significantly lower than the control tumors (Fig. 5c). The mice body weights were not significantly different between the three groups (Fig. 5d), neither did we notice any signs of apparent toxicities in the experimental mice.

At D7 and D14, one tumor of each group was isolated, and tumor tissues were achieved and analyzed. Testing circPRKCI expression, by qPCR, demonstrated that circPRKCI levels were significantly downregulated in tumors with circPRKCI shRNA (Fig. 5e). On the contrary, miR-545 levels were dramatically increased (Fig. 5f). Protein expression of miR-545 targets, E2F7 and RIG-1, was downregulated in circPRKCI-silenced tumors (Fig. 5g). Thus, in line with the in vitro findings, circPRKCI shRNA induced miR-545 accumulation, E2F7 and RIG-1 downregulation in subcutaneous A172 glioma tumors.

### circPRKCI shRNA inhibits orthotopic A172 glioma growth in mice

**Fig. 6:**
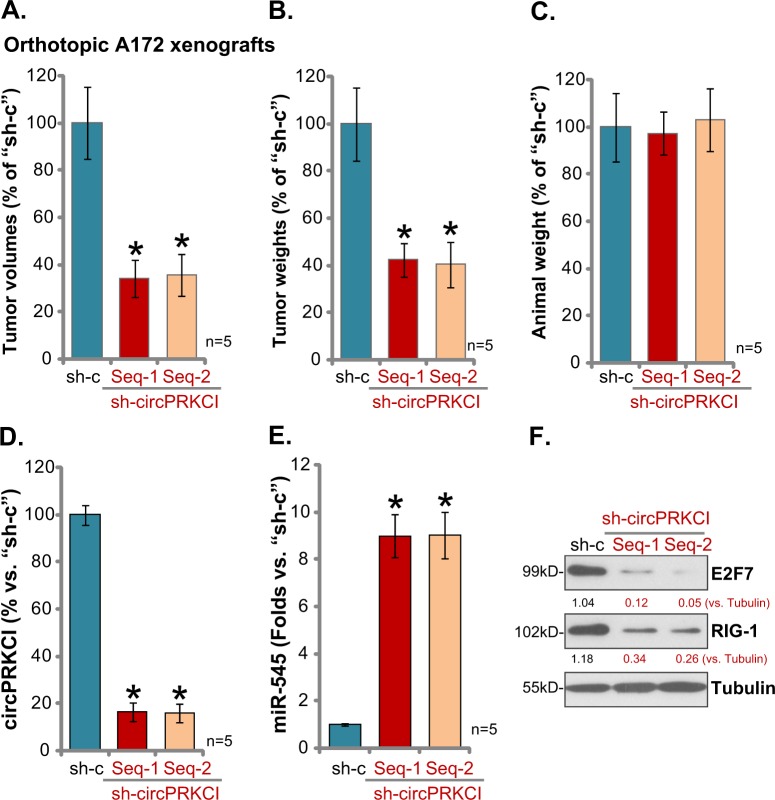
circPRKCI shRNA inhibits orthotopic A172 glioma growth in mice. Same number of A172 glioma cells, expressing circPRKCI shRNA (“Seq-1/Seq-2”) or scramble non-sense control shRNA (“sh-c”), were intracranially injected into the brain of SCID mice (5–6 week old, eight mice per group), after 21 days the animals were sacrificed and tumor volumes (**a**), tumor weights (**b**), and mouse body weights (**c**) recorded. Tumor tissue lysates were analyzed by qPCR and western blotting assays to test listed genes (**d**–**f**). E2F7 and RIG-1 protein expression was quantified (**f**). Error bars stand for mean ± standard deviation (SD, *n* = 5). *P < 0.05 vs. “sh-c” tumors

To further study a role of circPRKCI in glioma cell progression, orthotopic glioma xenografts were established. Exact same number of A172 cells (1 × 10^6^ cells of each mouse mouse) with circPRKCI shRNA (“Seq-1/Seq-2”) or scramble non-sense control shRNA (“sh-c”), were intracranially injected to the brain of SCID mice^19^. At day-21, with first mouse in the control tumor group showed the typical neurologic and sick symptoms, all groups were sacrificed, with tumors isolated. Results of tumor volumes (Fig. 6a) and the tumor weights (Fig. 6b) demonstrated that orthotopic A172 gliomas with circPRKCI shRNA grew significantly slower than the control tumors (Fig. 6a, b). Once again, the mice body weights were not significantly different between the three groups (Fig. 6c). Biochemical analyses of tumor tissue lysates confirmed that circPRKCI levels were significantly downregulated in the orthotopic A172 xenografts with circPRKCI shRNA (Fig. 6d), whereas miR545 levels significantly increased (Fig. 6e). miR-545 targets, E2F7 and RIG-1, were downregulated in circPRKCI-silenced orthotopic tumor tissues (Fig. 6f). Therefore, circPRKCI shRNA inhibited orthotopic A172 glioma growth in mice.

## Discussion

circRNAs are a large and conserved family of non-coding RNAs, generated from a non-canonical back splicing process, from a covalent bond between 5′ and 3′ ends of a single-stranded RNA^14,33^. Dysregulation of circRNAs has been detected in glioma cells, essential for cancer development and progression^12^. Our results suggest that circPRKCI is an oncogenic circRNA in glioma. Its expression is upregulated in human glioma tissues and in established/primary human glioma cells, but low in normal brain tissues and neurons/astrocytes. In A172 cells and primary human glioma cells, circPRKCI silencing, by targeted shRNA, potently inhibited cell growth, survival, proliferation, and migration, whiling inducing apoptosis activation. Conversely, exogenous overexpression of circPRKCI, by a lentiviral construct, promoted A172 cell progression. Furthermore, in vivo growth of subcutaneous and orthotopic A172 gliomas was significantly inhibited by circPRKCI silencing. These results suggest that circPRKCI could be an important and novel therapeutic target of glioma.

Recent studies have shown that miR-545 is a tumor-suppressive miR in human cancers^16,28,29^. Du et al., have shown that miR-545 can inhibit human lung cancer cells^29^. In human pancreatic ductal adenocarcinoma cells, miR-545 inhibits RIG-1 expression and cancer cell growth^28^. In lung cancer cells circPRKCI promotes cancer cell progression by sponging miR-545^16^. In this study, we show that miR-545 inhibition or knockout promoted A172 cell progression. Conversely, forced overexpression miR-545 inhibited A172 cell survival and proliferation. Therefore, miR-545 plays a tumor-suppressive role in human glioma cells.

Two primary miR-545 targets are RIG-1 and E2F7 in cancer cells^16,26,28,34^. RIG-I is an intracellular viral RNA sensor, whose activation could initiate host innate immune response to increase type I IFN production^35^. Recent studies have proposed a role of RIG-1 in cancer progression. For instance, Song et al., demonstrated that high RIG-I protein level in pancreatic ductal adenocarcinoma tissues is correlated with shorter survival^28^. Two cancer studies have discovered that miR-545 inhibits human cancer progression by targeting RIG-I^28,34^. E2F7 is a relatively novel transcription factor, regulating cell cycle by inhibiting expression of G1-S genes^36,37^. E2F7 could form a heterodimer with E2F1 and recruits the co-repressor C-terminal-binding protein (CtBP)^16^. Recent studies have implied that downregulation of E2F7 could induce cancer cell apoptosis^38,39^. Importantly, E2F7 could be an independent prognostic factor of gliomas, whose overexpression predicts poor prognosis in glioma patients^40^. Thus, E2F7 could be a novel therapeutic target of human glioma^40^. E2F7 is one key mRNA target of miR-545^16^. Here, in A172 and primary human glioma cells ectopic miR-545 overexpression significantly downregulated RIG-1 and E2F7, both were upregulated with miR-545 inhibition.

circPRKCI could sponge miR-545 and possible other tumor-suppressive miRNAs^16^. The results of the current study indicate that miR-545 is the primary target of circPRKCI in glioma cells. RIP assay results show that circPRKCI and miR-545 were both efficiently pulled down by anti-Ago2 antibody in A172 glioma cells. miR-545 levels were significantly increased in circPRKCI-silenced A172 cells, with its targets, *E2F7* and *RIG-1*, downregulated. Importantly, exogenous overexpression of miR-545 by a lentiviral construct potently inhibited A172 cell progression, mimicking circPRKCI shRNA-induced activity. Conversely, miR-545 inhibition, via LV-antagomiR-545, abolished circPRKCI silencing-induced anti-A172 cell activity. Significantly, miR-545 inhibition or knockout (by CRISPRC/Cas9 method) promoted A172 cell progression. Remarkably, neither circPRKCI shRNA nor circPRKCI overexpression was effective in the miR-545-KO A172 cells. In the circPRKCI-silenced subcutaneous and orthotopic A172 xenograft tumor tissues, miR-545 levels were significantly upregulated, correlating with downregulation of its targets, RIG-1 and E2F7. Finally, we show that in human glioma tissues and cells, circPRKCI upregulation correlates with miR-545 downregulation. These results indicate that circPRKCI promotes glioma cell progression possibly by sponging miR-545. miR-545 should be the direct target of circPRKCI in glioma cells.

## Conclusion

circPRKCI promotes human glioma cell progression possibly by inhibiting miR-545. Targeting circPRKCI-miR-545 cascade could be a novel strategy to inhibit human glioma.
